# Reliability and validity of the physical activity monitor for assessing energy expenditures in sedentary, regularly exercising, non-endurance athlete, and endurance athlete adults

**DOI:** 10.7717/peerj.9717

**Published:** 2020-08-24

**Authors:** Chun-Hao Chang, Yi-Ju Hsu, Fang Li, Yu-Tsai Tu, Wei-Lun Jhang, Chih-Wen Hsu, Chi-Chang Huang, Chin-Shan Ho

**Affiliations:** 1 Graduate Institute of Sports Science, National Taiwan Sport University, Taoyuan, Taiwan; 2 Department of Physical Medicine and Rehabilitation, Taipei City Hospital, Zhongxiao Branch, Taipei, Taiwan

**Keywords:** Wearable sensor, Heart rate reserve, Oxygen uptake, Athlete, Physical fitness, Energy expenditure

## Abstract

**Background:**

Inertial sensors, such as accelerometers, serve as convenient devices to predict the energy expenditures (EEs) during physical activities by a predictive equation. Although the accuracy of estimate EEs especially matter to athletes receive physical training, most EE predictive equations adopted in accelerometers are based on the general population, not athletes. This study included the heart rate reserve (HRR) as a compensatory parameter for physical intensity and derived new equations customized for sedentary, regularly exercising, non-endurance athlete, and endurance athlete adults.

**Methods:**

With indirect calorimetry as the criterion measure (CM), the EEs of participants on a treadmill were measured, and vector magnitudes (VM), as well as HRR, were simultaneously recorded by a waist-worn accelerometer with a heart rate monitor. Participants comprised a sedentary group (SG), an exercise-habit group (EHG), a non-endurance group (NEG), and an endurance group (EG), with 30 adults in each group.

**Results:**

EE predictive equations were revised using linear regression with cross-validation on VM, HRR, and body mass (BM). The modified model demonstrates valid and reliable predictions across four populations (Pearson correlation coefficient, *r*: 0.922 to 0.932; intraclass correlation coefficient, ICC: 0.919 to 0.930).

**Conclusion:**

Using accelerometers with a heart rate monitor****can accurately predict EEs of athletes and non-athletes with an optimized predictive equation integrating the VM, HRR, and BM parameters.

## Introduction

A balance between the energy expenditure (EE) and energy intake should be carefully considered for the performance of athletes (Zabriskie et al., 2019; Kumahara et al., 2020). Therefore, it is necessary to measure the EE of athletes during their daily life. In general, most athletes have higher EEs, reflecting their high-level physical activities and body composition (Ndahimana et al., 2017). Accordingly, compared with non-athletes, athletes are in greater nutritional demand (Ranchordas et al., 2013). Because the body mass of athletes is often precisely managed, their EE should be accurately assessed to predict the nutritional requirement. Underestimation of EE incurs inadequate nutrient intake, which impairs the musculoskeletal system and compromises the exercise performance, including running, jumping, and agility (Arieli & Constantini, 2012; Burrows et al., 2016; Mujika et al., 2018).

Grasping personal EE of an athlete helps a sports coach or trainer assess training loads required for the periodization or season training plans involving particular exercises. This information also provides recommendations for calorie uptake (Taylor et al., 2018). Although EE of various physical activities can be accurately predicted by indirect calorimetry and doubly labeled water (DLW) (Gao et al., 2012; Beltrame et al., 2016; Lee et al., 2016; Westerterp, 2017), these applications are usually limited due to high cost and requirements of special instruments. Therefore, inertial sensors, such as accelerometers, have been developed in recent years and been widely used in daily life, sports training, health care, and even movie and animation industries as a more economical and convenient way to measure EE (Dutta et al., 2018; Katapally et al., 2018; Taylor et al., 2018; Pavon et al., 2020). Accelerometer-based wearable devices estimate EE more efficiently because of their lower cost, uncomplex operation, and a possibility to track measure physical activity continuously for weeks (Shih, Ho & Shiang, 2014; Menai et al., 2017; Kim & Lochbaum, 2017; Dutta et al., 2018). However, factors contribute to the accuracy of EE estimation, including sensor designs, proprietary software with reliable equations (Price et al., 2017), types of exercises (e.g., hills, biking or muscle machine training) (Stec & Rawson, 2012; Herman Hansen et al., 2014; Kuo et al., 2018), and physiological properties (Brazeau et al., 2014).

The accelerometer-based sensor quantifies acceleration using one or three vertical axes to track continuous movements during a period. The measured values can be output into activity counts, steps, time spent at different physical activity intensities or cadences, and estimated EE predicted by equations (Yang, Gerhard & Barden, 2015; Larsen et al., 2017; Sirichana et al., 2017). In the development of the EE predictive equations, treadmill exercise tests and daily activity performance tests are usually considered to be factors of physical activities, and the EE predictive equations are mainly based on healthy adults in the general population. In previous studies, few EE predictive equations have been derived explicitly from particular populations, so their predicted EE would be inaccurate. At present, EE predictive equations have been developed for populations such as children or adolescents (Jimmy, Seiler & Maeder, 2013; Butte et al., 2014; Crouter, Oody & Bassett Jr, 2018), the elderly (Aguilar-Farias et al., 2018), and even wheelchair users (Hiremath & Ding, 2011; Hiremath et al., 2012). However, EE predictive equations have rarely been designed for athletes so far.

Athletes have well-developed muscular systems and even larger organs (such as the liver, heart, and kidneys) (Miyauchi et al., 2013). Since more muscles consume more energy according to the oxygen uptake (Armstrong & McManus, 2011; Farinatti & Castinheiras Neto, 2011), athletes have a higher resting metabolic rate (RMR) and total energy expenditure (TEE) than non-athletes (Ribeyre et al., 2000; Kim et al., 2015; Ndahimana et al., 2017). However, despite applications of wearable devices for measuring activity load (Ritchie et al., 2016; Stiles et al., 2018) and estimate EE (Yang, Gerhard & Barden, 2015; Larsen et al., 2017; Sirichana et al., 2017), variations between athletes and non-athletes are rarely considered. Currently, the Japan Institute of Sports Sciences (JISS) calculates estimate energy requirement (EER) by multiplying RMR by 2.0 for athletes in ball games or by 2.5 for endurance runners indiscriminately (Koshimizu et al., 2012; Japan Institute of Sports Sciences, 2016; Park, 2019). However, EE differs even within the same sport, depending on the amount of training or load training of competition, and the variations are more remarkable between sports types (Park, 2019).

If athletes adopt accelerometers with an EE predictive equation derived merely from the general population, their EE may be underestimated. Some approaches attempt to calibrate estimate EE simply by including heart rates (HR) as a parameter reflecting the exercise intensity (Domene & Easton, 2014; Park et al., 2017; Lu et al., 2018; Kuo et al., 2018). However, it, on the other hand, introduces biases to estimate EE because HR is susceptible to physical fitness and psychological factors, such as excitement and nervousness (Patrik Johansson et al., 2006). Previously, we demonstrated the heart rate reserve (HRR) to be a preferable parameter that considers exercise intensity while standardizing individual variations (Chang et al., 2019). In light of this, this study included HRR to be an important indicator for calibrating the physical activity levels among different groups to improve the accuracy of EE estimation. In this study, we modified the model for the general population by including HRR parameters and developed new EE predictive equations for athletes and non-athletes.

## Materials & Methods

### Study Design

In this research, we used indirect calorimetry and an accelerometer with an HR monitor to measure and predict the EE of athletes and non-athletes. In the EE predictive equations, various physical activity level must be satisfied to make it more applicable. The physical activity level can be directly reflected through HRR (Karvonen, Kentala & Mustala, 1957; Djaoui et al., 2017). Therefore, this study used the HRR parameters as a correction factor to increase the accuracy of the estimate EE. We modified the EE predictive equation used for the general adult population to develop one applicable to the athlete population. From these measurements in this study, linear regression equations were determined for the level each physical activity based on average accelerometer vector magnitudes (VM), body mass (BM), and HRR parameters, and the criterial EE measured by indirect calorimetry. A laboratory experiment was implemented to support the development of the modified model in this study and evaluate the existing model. This research procedure was approved by the Institutional Review Board of Fu Jen Catholic University (New Taipei City, Taiwan) (reference number: C106056). All participants completed informed consent forms before starting the experimental test.

### Participants

Athletes and non-athletes were recruited using an open, independent, and random method by posting advertisements in public spaces and campuses. They were required to complete an International Physical Activity Questionnaire (IPAQ group, 2018) and categorized into one of four groups, each of which contained 30 participants. The non-athlete participants were separated into two groups: the sedentary group (SG, male: 40.0%, female: 60.0%) comprised participants who did not exercise regularly and spent most of their time sitting or lying down, while the exercise-habit group (EHG, male: 46.7%, female: 53.3%) was composed of those who regularly exercised with moderate- to vigorous-intensity physical activity at least three days a week for 30 to 60 min according to the guidelines of the American Heart Association (AHA, 2018). The athletes were further classified in two groups: the non-endurance group (NEG, male: 56.7%, female: 43.3%) comprised athletes that focused on strength/speed type sports including sprint races (the 100 m, 200 m, and 400 m) and throwing sports (shot put and javelin), had ever participated in the National Games within two years, and were routinely trained for 27.2 ± 2.2 h in five days a week following a moderate- to a vigorous-intensity specific training program; the endurance group (EG, male: 63.3%, female: 36.7%) was composed of athletes that focused on middle-distance (800 m, 1,500 m, and 3,000 m) or long-distance races (longer than 3,000 m) in track and field, had ever participated in the National Games within two years, and were routinely trained for 27.8 ± 1.5 h in five days a week following a moderate- to vigorous-intensity specific training program.

Those who had any exercise contraindications, currently took medications that affect metabolic rates, were diagnosed with any cardiovascular disorders by physicians, or had any factors that could affect safety completion of the test procedures were excluded. Participants were restricted from diet, caffeine, and exercise for 4 h before the test (Lyden et al., 2011) and slept for at least 8 h before the day of the test to avoid variance in metabolic rates and heart rates. The experiments were performed during day time, from 6:00 am to 5:00 pm. Participants were required to complete a one-hour test wearing a monitoring system. The room temperature was controlled at 23 °C to minimize the effects of temperature during exercise.

### Anthropometric Measurements and Body Composition

Anthropometric measurements and body composition were assessed through standard procedures (Miller, Chambers & Burns, 2016; Ndahimana et al., 2017). Participants’ body mass index (BMI) and body composition were measured with the InBody^^®^^ 570 Body Composition Analyzer (Biospace, Inc. Seoul, Korea), with additional inputs of their age and height measured by the height measurement equipment H900 (NAGATA Scale Co., Ltd. Tainan, Taiwan). The InBody^^®^^ 570 serves as a reliable method to estimate body composition, including skeletal muscle mass, percentage of body fat, and resting metabolic rate (RMR), with a multi-frequency bioelectrical impedance analyzer (Miller, Chambers & Burns, 2016). All participants wore lightweight sportswear, except shoes and socks, and any external metal items were removed.

### Measurement of EE by indirect calorimetry and accelerometer method

For the metabolic criterion measure (CM), we performed an indirect calorimetry method with a cardiopulmonary exercise testing system (Vmax Encore 29 System, VIASYS Healthcare Inc, Yorba Linda, CA). The mouth and nose of participants were covered by a mask (Hans Rudolph Inc., Kansas City, MO, USA) attached to a sampling tube that was connected to a digital flow sensor measuring the breath-by-breath tidal volume and the composition of the oxygen uptake (VO_2_) and carbon dioxide output (VCO_2_).

In the accelerometer method, we used the ActiGraph GT9X-Link (Actigraph Corporation, Pensacola, FL, USA, firmware version 1.7.1), a small (3.5 × 3.5 × 1 cm) and light (∼14 g) tri-axial accelerometer, to collect tri-axial activity data. Before the test, we used the ActiLife6 software (version 6.12.1, ActiGraph, Cary, NC, USA) to initialize the GT9X-Link and configured a compatible chest strap for the Polar H10 Heart Rate Monitor (Polar Electro Oy, Finland). In this research, the sampling frequency of GT9X-Link was 30 Hz with a lower frequency extension filter, and the data of activity count and HR were collected in 10-second epochs using ActiGraph cut-points defined by Sasaki, John & Freedson (2011) (sedentary, 0–200; light, 201–2690; moderate, 2691–6166; vigorous, 6167–9642 counts per minute). According to ActiGraph’s User Manual, the GT9X-Link was fastened around the waist with the adjustable elastic band on the participant’s right hip in line with the midaxillary line. Participants were assigned to a treadmill, including five walking or running speeds in random order. Throughout the test, the system simultaneously and continuously recorded the VO_2_ and VCO_2_ via indirect calorimetry, HR, and accelerometer count. The clock times of the Vmax system and GT9X-Link were synchronized to the time of the ActiLife6 software’s computer.

### Treadmill test

At the onset of the experiments, resting heart rates (HR _rest_) was determined by detecting the lowest HR recorded during the last 5 min in 20-minute sitting in the lab environment (Lounana et al., 2007). Participants were then required to perform walk/run tests at paces of 4.8, 6.4, 8.0, 9.7, and 11.3 km/h on a treadmill. Each speed set lasted 3 min, with 2-minute rest intervals between sets (test method adapted from Tudor-Locke, Barreira & Schuna Jr, 2015). In any activity sets, tests would be immediately terminated with data excluded from analyses if the heart rate of the participants exceeded the safe range, 220 bpm minus the participant’s age), or if they could not complete the exercise test safely, for example not unable to keep up with the treadmill speed.

### Data analysis

All 120 participants completed the exercise tests. The data from the indirect calorimetry (Vmax system), HR monitor (Polar H10), and an accelerometer (ActiGraph GT9X-Link) were output to Microsoft Excel (Excel version in Microsoft Office 2013 for Windows). The Vmax systems and HR monitors recorded the parameters and output records in 10-second intervals, synchronized with ActiGraph GT9X-Link accelerometer. All data were processed, according to Lyden et al. (2011). The first 120 s in each speed setting were trimmed to ensure that the participants achieved stability in movement under the exercise intensity. The VO_2_ and VCO_2_ were calculated to estimate criterion measure EE (CMEE) by the Weir equation: CMEE(kcal∕min) = 3.491 × *VO*_2_(L∕min) + 1.106 × *VCO*_2_(L∕min) (Weir, 1949). The GT9X-Link data (GT9X EE) were analyzed by ActiLife6 software, and the EE was calculated using the equation of the Freedson’s VM3 Combination (2011): GT9XEE(kcal∕min) = 0.001064 × V M + 0.087512 × BW(kg) − 5.500229 (ActiGraph, 2011), where the 3D $\mathrm{V M}=\sqrt{(\text{axis} 1)^{2}+(\text{axis} 2)^{2}+(\text{axis} 3)^{2}}$ was analyzed by 10-s epoch lengths in ActiLife6 software. All EE values were divided by the body mass to be for gender-standardized adjustments and presented as kcal/min/kg. HRR = HR_*max*_ − HR_*rest*_, which indicates the difference between HR_max_ and HR_rest_ at each treadmill speed, where HR_rest_ is the pre-test measure of resting HR, and HR_max_ is the highest HR measured for each phase of the test.

### Statistical analysis

All data are presented as the means ± standard deviations (SD). The one-way ANOVA test was employed to compare the differences in primary data and body composition among the four groups. To analyze the EE differences among the four groups at each speed, multivariate analysis of variance (MANOVA) was used, followed by the Games-Howell post hoc test. To investigate the EE difference between the two systems of measurement, CMEE and GT9X EE, we adopted paired t-tests and calculated Cohen’s d effect size (ES) and mean absolute percentage error (MAPE= {[—(predicted value - actual value)—/actual value] * 100}/n). Linear regression was used for modification of the EE prediction model with VM, body mass (BM), and HRR, and Train/Test Split was applied for cross-validation, in which data were randomly split into training (70%) and test (30%) subsets. Then, the validity and reliability of the EE estimation were further evaluated by the criterion analysis, namely the Pearson correlation coefficient (*r*) and the intraclass correlation coefficient (ICC; two-way mixed models; absolute agreement), respectively. The statistical software IBM SPSS Statistics version 20 (IBM Corp., New York, NY, USA) was used for statistical analysis. The significance level was set to *α* = 0.05.

## Results

### Anthropometry and body composition

120 subjects completed the exercise test safely. The compositions of groups did not differ in age (*p* = 0.118) or height (*p* = 0.078), but diverged in body mass (BM) (*p* = 0.057), body mass index (BMI) (*p* = 0.010), skeletal muscle mass (*p* = 0.005), percentage of body fat (*p* < 0.001), and resting metabolic rate (RMR) (*p* = 0.011) Table 1. Specifically, according to post-hoc results, in the BM section, EG is significantly lower than SG (59.8 ± 8.0 kg vs 67.2 ± 13.9 kg; *p* = 0.034), and NEG (59.8 ± 8.0 kg vs 68.7 ± 16.9 kg; *p* = 0.011). In the BMI section, NEG is significantly higher than EG (23.3 ± 4.7 kg/m^2^ vs 20.8 ± 2.1 kg/m^2^; *p* = 0.008), EG is significantly lower than SG (20.8 ± 2.1 kg/m^2^ vs 23.7 ± 3.5 kg/m^2^; *p* = 0.002), and EHG (20.8 ± 2.1 kg/m^2^ vs 23.1 ± 3.3 kg/m^2^; *p* = 0.014). In the skeletal muscle mass section, both SG (26.6 ± 5.7 kg vs 32.1 ± 7.1 kg; *p* < 0.001) and EHG (28.4 ± 6.0 kg vs 32.1 ± 7.1 kg; *p* = 0.015) are significantly lower than NEG. In the percentage of body fat section, NEG is significantly higher than EHG (16.3 ± 5.4% vs 20.2 ± 6.6%; *p* = 0.019); and EG is significantly lower than SG (8.4 ± 3.8% vs 19.1 ± 8.5%; *p* < 0.001), EHG (8.4 ± 3.8% vs 20.2 ± 6.6%; *p* < 0.001), and NEG (8.4 ± 3.8% vs 16.3 ± 5.4%; *p* < 0.001). In the resting metabolic rate (RMR) section, both SG (1414.1 ± 200.7 kcal/day vs 1591.1 ± 250.6 kcal/day; *p* = 0.001) and EHG (1464.2 ± 206.9 kcal/day vs 1591.1 ± 250.6 kcal/day; *p* = 0.019) are significantly lower than NEG.

**Table 1:** Anthropometry and body composition characteristics of participants.

	SG	EHG	NEG	EG	*p*	Range
Age (years)	21.9 ± 1.9	21.7 ± 1.6	21.1 ± 1.7	20.9 ± 1.7	0.118	18.0–26.0
Height (cm)	166.9 ± 8.1	167.3 ± 8.5	171.2 ± 7.7	170.0 ± 5.8	0.078	150.0–182.0
Sex	12 males 18 females	14 males 16 females	17 males 13 females	19 males 11 females		
Body mass (kg)	67.2 ± 13.9^b^	64.6 ± 12.6	68.7 ± 16.9^b^	59.8 ± 8.0	0.057	46.0–133.6
BMI (kg/m^2^)	23.7 ± 3.5^b^	23.1 ± 3.3^b^	23.3 ± 4.7^b^	20.8 ± 2.1	0.010	17.0–38.1
Skeletal muscle mass (kg)	26.6 ± 5.7^a^	28.4 ± 6.0^a^	32.1 ± 7.1	29.1 ± 4.5	0.005	18.0–51.4
Percentage of body fat (%)	19.1 ± 8.5^b^	20.2 ± 6.6^a^^b^	16.3 ± 5.4^b^	8.4 ± 3.8	<0.001	2.7–35.8
RMR (kcal/day)	1414.1 ± 200.7^a^	1464.2 ± 206.9^a^	1591.1 ± 250.6	1490.0 ± 160.0	0.011	1102.0–2295.0

**Notes.**

a Significantly different from NEG, *p* < 0.05.

b Significantly different from EG, *p* < 0.05.

Values are reported as the mean ± standard deviation.

SG sedentary group EHG exercise habit group NEG non- endurance group EG endurance group BMI body mass index RMR resting metabolic rate

*p*-value was calculated from one-way ANOVA test among the four groups

### CMEE and GT9X EE accelerometer data

**Figure 1:**
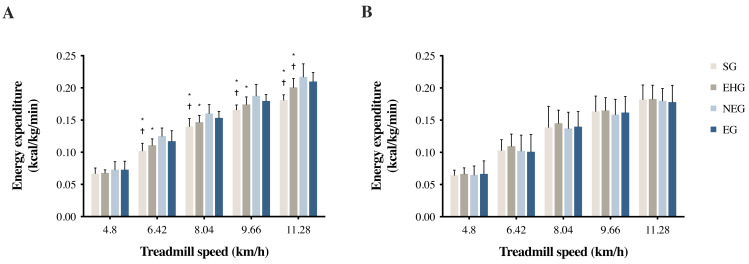
(A) CMEE and (B) GT9X EE during treadmill walking/running tests at different speeds. CMEE, criterion measure of energy expenditure measured by the indirect calorimetry (Vmax system). GT9X EE, energy expenditure predicted by the accelerometer (ActiGraph GT9X-Link). * Significantly different from NEG, *p* < 0.05. † Significantly different from EG, *p* < 0.05.

The results of the CMEE and GT9X EE accelerometer data analyzed by the MANOVA indicated a significant difference in CMEE measurements among the four groups (*p* < 0.001) (Fig. 1). Except for the walking speed of 4.8 km/h, which showed no significant difference in CMEE among the four groups (*p* = 0.116), the CMEEs were significantly higher in the NEG and EG than in the SG and EHG (*p* < 0.001) at the running speeds (6.4, 8.0, 9.7, and 11.3 km/h). However, the results of the GT9X EE on each treadmill speed test showed no differences among the four groups (*p* > 0.05). Table 2 lists the CMEE and GT9X EE values of the four groups from the treadmill test, as well as the differences in the paired *t*-test, ES, MAPE, and ICC. When individuals in the SG and EHG ran at a pace of 11.28 km/h, the measurement results of these two systems reached a significant difference (SG: *p* = 0.013; EHG: *p* < .001) compared with the other speeds (*p* > 0.05). The results also showed very minor differences in effect size (SG: 0.20 to 0.53; EHG: 0.12 to 0.19) and MAPE (SG: 4.3%; EHG: 3.0%) and good to excellent reliability (ICC = 0.851 and 0.915, respectively). However, the CMEE measurements in the NEG (*p* < 0.012) and EG (*p* < 0.019) were significantly higher than the GT9X EE results for all treadmill speed tests, with larger ES (NEG: 0.59 to 1.90; EG: 0.46 to 2.21) and MAPE (NEG: 15.7%; EG: 11.2%), and the lowest reliability was found in the NEG (ICC = 0.778).

### Comparison between modified models and GT9X EE

**Figure 2:**
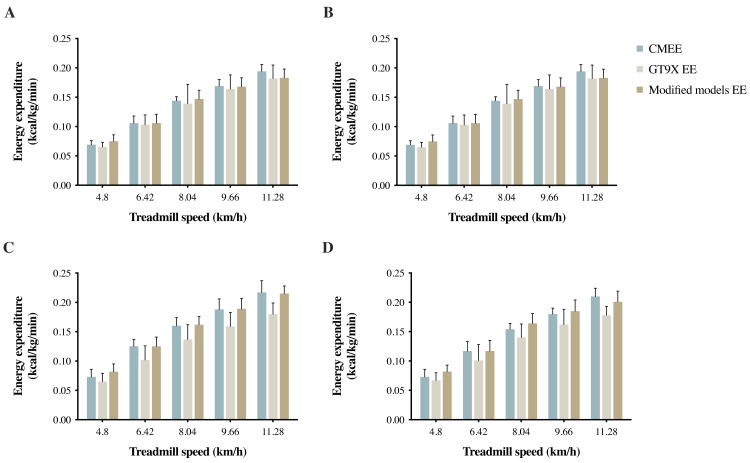
(A) SG, (B) EHG, (C) NEG, and (D) EG measured EE by Vmax system (CMEE), and estimated EE GT9X EE (Freedson VM3 Combination) and modified models EE by GT9X in treadmill tests.

Table 3 shows the results of linear regression with cross-validation of EE predictive models composed of VM activity counts, body mass (BM), and HRR. The modified models from the four groups all had high values of the coefficient of determination (*R*^2^ = 0.851 to 0.869) and a very small standard error of the estimate (SEE). The CMEE, GT9X EE, and modified models EE results are shown in Fig. 2. Table 4 lists the validity analysis (*r*) and the reliability analysis (ICC) for calculating the EE and CMEE among the different groups. The *r* and ICC values of the modified models were both greater than those of the Freedson VM3 Combination equation (*r* = 0.922 to 0.932, good correlation; ICC = 0.919 to 0.930, high ICC). The main difference between the Freedson VM3 combination equation and the modified models is the HRR factor. From the above results, it appears that the HRR can be used as a fairly accurate predictor within various physical activity levels populations to improve the reliability and validity of predictive values, as well as the accuracy of the predictive model.

## Discussion

This research explored the effect of accelerometer outputs on EE estimation in populations with different levels of physical activity and established suitable predictive equations. The cross-validations revealed that the correlation differences of linear regressions for four groups were between 70% and 30% were from 0.001 to 0.026, and all correlations were 0.928 to 0.936. According to criterion measurements, EEs of various populations under several speed tests are different (*p* < 0.05). However, accelerometers alone were unable to distinguish EEs between various groups (*p* > 0.05). Adopting the Freedson’s VM3 Combination (2011) EE predictive equation, we could make an accurate prediction in the populations with a sedentary lifestyle and exercise habits (SG: MAPE = 4.3%, ICC = 0.851; EHG: MAPE = 3.0%, ICC = 0.915), but the EEs were still underestimated in the athlete population (NEG: MAPE = 15.7%, ICC = 0.778; EG: MAPE = 11.2%, ICC = 0.837). Therefore, if the equation is applied to predict an athlete’s EE, the underestimate of EEs may result in insufficient nutrient intake and thereby a negative energy balance. In previous studies, HRR was a satisfactory parameter to adjust for an individual’s physical fitness level (Patrik Johansson et al., 2006). In this research, the reliability of the EE estimation among the four groups was improved by including the HRR parameters in the predictive equation. The modified models were valid and reliable (SG: *r* = 0.922, ICC = 0.919; EHG: *r* = 0.932, ICC = 0.930; and NEG: *r* = 0.929, ICC = 0.927; EG: *r* = 0.930, ICC = 0.927, respectively). Since most canonical EE predictive equations mainly consider accelerometer outputs, body mass (BM), and constants, they fail to distinguish individuals with different physical activity levels. The improvement in the EE estimation, considering the HRR, accelerometer outputs, and BM, allowed us to tell apart the differences in physical activity levels among various groups.

**Table 2:** Comparison of EE measured by indirect calorimetry (Vmax system) and EE estimated by GT9X EE, and HR in five treadmill walking/running tests.

Group	Treadmill speed (km/h)	CMEE (kcal kg^−1^ min^−1^)	GT9X EE (kcal kg^−1^ min^−1^)	ES	MAPE (%)	ICC	HRrest(BPM)	HRmax (BPM)
SG	4.80	0.069 ± 0.007	0.065 ± 0.008	0.53	4.3	0.851	81.34 ± 8.97	108.59 ± 13.60
	6.42	0.106 ± 0.012	0.103 ± 0.017	0.20				131.29 ± 14.24
	8.04	0.144 ± 0.007	0.139 ± 0.033	0.21				157.06 ± 11.01
	9.66	0.169 ± 0.011	0.164 ± 0.024	0.27				172.71 ± 12.17
	11.28	0.194 ± 0.012	0.182 ± 0.023^a^	0.65				183.37 ± 13.64
EHG	4.80	0.068 ± 0.005	0.067 ± 0.009	0.14	3.0	0.915	75.96 ± 7.02	101.26 ± 8.88
	6.42	0.111 ± 0.010	0.109 ± 0.019	0.13				124.14 ± 11.19
	8.04	0.147 ± 0.011	0.145 ± 0.020	0.12				146.11 ± 12.96
	9.66	0.168 ± 0.011	0.165 ± 0.020	0.19				163.12 ± 12.06
	11.28	0.201 ± 0.014	0.183 ± 0.022^a^	0.98				176.39 ± 11.97
NEG	4.80	0.073 ± 0.013	0.065 ± 0.014^a^	0.59	15.7	0.778	76.81 ± 8.03	98.53 ± 11.46
	6.42	0.125 ± 0.012	0.102 ± 0.024^a^	1.21				120.08 ± 10.79
	8.04	0.160 ± 0.014	0.137 ± 0.025^a^	1.14				138.99 ± 11.51
	9.66	0.188 ± 0.018	0.159 ± 0.024^a^	1.37				153.81 ± 12.97
	11.28	0.217 ± 0.020	0.180 ± 0.019^a^	1.90				169.62 ± 12.24
EG	4.80	0.073 ± 0.013	0.067 ± 0.013^a^	0.46	11.2	0.837	66.93 ± 9.20	86.81 ± 12.94
	6.42	0.117 ± 0.016	0.101 ± 0.027^a^	0.72				104.36 ± 14.35
	8.04	0.154 ± 0.010	0.140 ± 0.023^a^	0.79				122.82 ± 15.89
	9.66	0.180 ± 0.010	0.162 ± 0.026^a^	0.91				134.41 ± 17.45
	11.28	0.210 ± 0.014	0.178 ± 0.015^a^	2.21				145.36 ± 18.07

**Notes.**

a Significantly different from CMEE, *p* < 0.05.

Values are reported as the mean ± standard deviation.

SG sedentary group EHG exercise habit group NEG non-endurance group EG endurance group CMEE criterion measure energy expenditure GT9X ActiGraph GT9X-Link accelerometer ES Effect size (Cohen’s d); Mean absolute percentage error (MAPE) = [(predicted value - actual value)/actual value] * 100/n ICC intraclass correlation coefficient HRrest the pre-test measure of resting HR HRmax the highest HR measured for each phase of the test BPM beats per minutes

**Table 3:** Modified models to predict EE (kcal kg^−1^ min^−1^) from VM, BW, and HRR.

Group	Coefficients	Unstandardized coefficients (B)	Standardized coefficients (*β*)	*p*	VIF	*R*^2^	SEE	*F*	D-W test
SG	Constant	0.039438		<0.001		0.851	0.018	278.39	1.827
	VM	0.000004	0.255	<0.001	2.995				
	BM	−0.000117	−0.035	<0.001	1.004				
	HRR	0.001077	0.701	0.279	2.990				
EHG	Constant	0.037952		<0.001		0.869	0.018	321.95	2.046
	VM	0.000007	0.396	0.072	3.309				
	BM	−0.000219	−0.057	<0.001	1.096				
	HRR	0.000954	0.578	<0.001	3.292				
NEG	Constant	0.066446		<0.001		0.863	0.020	306.49	1.309
	VM	0.000007	0.343	<0.001	2.653				
	BM	−0.000558	−0.167	<0.001	1.097				
	HRR	0.001240	0.639	<0.001	2.717				
EG	Constant	0.028271		0.026		0.864	0.019	308.59	1.245
	VM	0.000012	0.603	<0.001	2.942				
	BM	−0.000144	−0.023	0.449	1.019				
	HRR	0.000771	0.371	<0.001	2.940				

**Notes.**

Modified models were developed on 70% of samples for modeling and cross-validated on the remaining 30% samples.

VM vector magnitudes BM body mass in kg HRR heart rate reserve VIF variance inflation factor R2 coefficient of determination which was measured from linear regression models SEE standard error of estimate D-W test, Durbin-Watson test

**Table 4:** Validity and reliability analysis of the predicted EE in models and CMEE in different groups.

Group	Freedson VM3 Combination		Modified models	
	*r*	ICC	*r*	ICC
SG	0.858	0.851	0.922	0.919
EHG	0.925	0.915	0.932	0.930
NEG	0.875	0.778	0.929	0.927
EG	0.917	0.837	0.930	0.927

**Notes.**

SG sedentary group EHG exercise habit group NEG non-endurance group EG endurance group r Pearsons correlation coefficient ICC intraclass correlation coefficient

When the speed (or exercise intensity) stayed the same, the CMEEs of both athlete groups were higher than those of the non-athlete groups, while the NEG had the highest value. In response to the speed (or exercise intensity), the EEs of all the four groups showed an analogous trend. Only in the low-intensity exercise was no difference in EE found between athlete groups and non-athlete groups. The results of this research are consistent with those of previous studies, suggesting that resting metabolic rate (RMR) and total energy expenditure (TEE) are higher in athletes than in non-athletes (Ribeyre et al., 2000; Kim et al., 2015; Ndahimana et al., 2017). The study of Petridou, Lazaridou & Mougios (2005) compared the TEEs of 14 male endurance athletes with those of non-athletes and found that the mean TEE of athletes was 3,895 ± 600 kcal/day, while that of non-athletes was 2,722 ± 475 kcal/day (*p* <0.05). Regarding resting energy expenditure (REE), the mean value of athletes was also higher than that of non-athletes (1,407.3 ± 170.2 kcal/day vs. 1,259.1 ± 105.6 kcal/day, respectively). The study of Ndahimana et al. (2017) compared the TEEs of college female tennis players and non-athletes (age: 19 to 24 years), and the mean TEE of tennis players was 2,780.3 ± 429.5 kcal/day, while that of non-athletes was 2,012.3 ± 160.5 kcal/day (*p* = 0.001). It is worth noting that although the metabolism varies between genders, the differences of EE flatten when normalized by body mass (BM) (from kcal/min to kcal/kg/min) (Loftin et al., 2010; Jagim et al., 2019). Therefore, we performed BM normalization for gender-standardized adjustments to rule out the gender effects.

On the other hand, an accelerometer outputs estimate EE by subsequent calculations of VM activity counts generated during exercise. Due to sensor characteristics, the accelerometer failed to identify the differences in physiological metabolism among various populations with different levels of physical activity or might cause overestimation or underestimation of EE based on different exercise types or intensities (i.e., cycling, uphill/downhill exercise, etc.) (Terrier, Aminian & Schutz, 2001; Patrik Johansson et al., 2006; Tarp, Andersen & Østergaard, 2015; Kuo et al., 2018; Ho et al., 2019). In other words, under the same exercise intensity, VM activity counts did not change with different user groups or in certain situations. The inability to precisely identify the physical activity level of each user likely biased the results of EE estimation. In our research, it was found that the various speed measurements of the GT9X EE in the athlete groups were greatly underestimated (*p* < 0.05) and had large differences in effect size (NEG: 0.59 to 1.90; EG: 0.46 to 2.21). Since the current EE predictive equation using accelerometers is mostly applied to the healthy adult population (Crouter, Clowers & Bassett Jr, 2006; Lyden et al., 2011; Sirichana et al., 2017), the EE predictive equation that is most widely used is more suitable for the general population than for athletes. In this research, no significant differences in the results of GT9X EE and criterion measurements (*p* > 0.05) were observed in the non-athlete groups between speed tests (except for the speed of 11.28 km/h), and very few differences were found in effect size (SG: 0.20 to 0.53; EHG: 0.12 to 0.19); the measurements demonstrated good to excellent reliability. Because the exercise intensity at the treadmill speed of 11.28 km/h is already in vigorous-intensity exercise for non-athletes (i.e., depending on fitness level), oxygen uptake will increase dramatically to compensate energy expenditure. However, such changes in the physiological reactions are not easily measured by the accelerometer. Altogether, the applications of accelerometers are limited to the general population within a certain range of exercise intensities. Therefore, without considering the different conditions of body composition or physical activity levels of various populations, this type of non-standard measurement method (i.e., physical activity sensors) may create different levels of bias.

The majority of body movements involve contractions of skeletal muscles, which require sufficient energy for proper function. Increases in muscle mass create a greater need for energy as well as more gas exchange. The results of this research also revealed that when the skeletal muscle mass of an athlete is greater than that of a non-athlete (*p* < 0.05), the EE, as well as the RMR, of the athlete during exercise is much higher than that of the non-athlete (*p* < 0.05). It was also confirmed in a study by Ndahimana et al. (2017) that in comparison to the non-athlete group, athlete participants had significantly higher values for skeletal muscle mass (23.4 ± 2.3 kg vs. 19.1 ± 1.4 kg), and total energy expenditure adjusted for fat-free mass (65.3 ± 5.2 kcal/kg/day vs. 56.4 ± 2.0 kcal/kg/day, *p* = 0.001). It can be seen that athletes have more considerable energy expenditure than the sedentary participants during exercise. During movement, to meet the metabolic requirements of skeletal muscles, the autonomic nervous system controls the heart rates and expansion of blood vessels through a complex dynamic process (Fisher, Young & Fadel, 2015; Dong, 2016; Chen et al., 2017). In line with a large number of studies in recent years, VO_2_ and HR are closely related, and there is a linear correlation between them. Therefore, changes in HR can be used as a basis for the assessment of exercise intensity.

For this reason, research has suggested that an accelerator-based EE monitor with an HR tracking device can increase the accuracy of EE estimation. Brage et al. (2015) indicated that research to estimate EE accurately for energy balance and metabolic disorders was fundamental. In that study, 23 women and 23 men (ages 22–55 years) were enrolled. The researchers used accelerometer and HR data to estimate the EE, and they also corrected with the EE measured by doubly labeled water (DLW). The results showed that integrating the accelerator with an HR function and criterion measurements produced good validity, with biases <5% and high correlations. Kuo et al. (2018) integrated HR and accelerator parameters, demonstrating that exercise-related changes in HR (△HR) improved the accuracy of the EE predictive equation during walking uphill of participants. Chang et al. (2019) also corrected the accelerator-based traditional empirical EE formulas by using HR and HRR as compensation factors while participants were walking/running uphill and revealed that the HRR outperformed HR in the adjustment of physical intensity. Since the results of our research are consistent with the results of previous studies, raw HR may likely be unstable due to an individual’s physical fitness level or psychological factors, which could affect the accuracy of EE estimation. By using HRR as a compensation factor, the reliability of EE estimation in populations with different levels of physical activity can be improved, resulting in a predictive value closer to the actual value. The error rates of EE and CMEE calculated from the modified models were SG: 3.6%, EHG: 4.5%, NEG: 3.2%, and EG: 5.2%. Compared with the results of GT9X EE, the error rates of EE estimation were greatly decreased in the athlete groups (NEG: 15.7% to 3.2%, EG: 11.2% to 5.2%).

It is crucial to accurately estimate the EE of an athlete in there training and daily life. When the training load is adjusted, maintaining the overall energy balance by ensuring a balance between EE and energy intake plays a crucial role in improving the performance of an athlete. Inaccurate EE estimation may lead to insufficient nutrient intake, increase the risk of lower lean tissue mass, affect athletic performance, or even cause a variety of adverse health effects (Arieli & Constantini, 2012; Burrows et al., 2016). Besides, HRR, representing the difference between the maximum heart rate and the resting heart rate, can be used to standardize the excessive gap of resting heart rate caused by individual differences in physical activity levels and can be used as the basis for the estimation of EE and exercise intensity. The results revealed that HRR could be considered as a factor in EE predictive equations to improve the reliability and validity of EE prediction among the four groups in the study. In particular, there was a significant improvement in EE prediction among non-endurance athletes. In summary, we propose predictive equations by including HRR parameters and adjusting the coefficients, so that athletes and non-athletes can use their customized predictive equations to improve accuracy. The results of the study confine our discussion to the young athlete and non-athlete adults. Note that we designed the EE equations for healthy adults, so they are inapplicable to other populations, such as children, the elderly, and populations with specific diseases.

## Conclusions

Vector magnitude detected by accelerometers, HRR, and body mass provided a practical method to estimate EE. Although some advanced equipment and methods can estimate EE more accurately, the use of accelerators also provides acceptable EE estimation and more feasible for most populations. In the current study, the Actigraph tended to underestimate the EE during walking/running treadmill tests, especially athletes. Excitingly, the method proposed in this study successfully improved the accuracy of athletes’ EE estimates. We hope that it can be applied to the EE monitoring of athletes in the training process in the future. To expand the practical applications, participants of different physical activity levels should be included in the future to evaluate this method. It should be possible to obtain more accurate predictions of EE by using the rational coefficients of the predictive equations.

##  Supplemental Information

10.7717/peerj.9717/supp-1 Supplemental Information 1 The raw data shows all groups’ energy expenditure measured by indirect calorimetry and accelerometer, and also showing accelerometer output, heart rate reserve, and Inbody outputs

## Additional Information and Declarations

### Data Availability

The following information was supplied regarding data availability: The raw measurements are available as a Supplemental File.
